# Green and Scalable Fabrication of Sandwich-like NG/SiO_x_/NG Homogenous Hybrids for Superior Lithium-Ion Batteries

**DOI:** 10.3390/nano11092366

**Published:** 2021-09-11

**Authors:** Guilong Liu, Yilin Wei, Tiantian Li, Yingying Gu, Donglei Guo, Naiteng Wu, Aimiao Qin, Xianming Liu

**Affiliations:** 1 Key Laboratory of Function-Oriented Porous Materials of Henan Province, College of Chemistry and Chemical Engineering, Luoyang Normal University, Luoyang 471934, China; glliu@tju.edu.cn (G.L.); yilinwei27@163.com (Y.W.); Lt248861494@163.com (T.L.); gy17335979115@163.com (Y.G.); gdl0594@163.com (D.G.); wunaiteng@gmail.com (N.W.); 2 Key Laboratory of New Processing Technology for Nonferrous Metal & Materials, Guangxi Key Laboratory of Optical and Electronic Materials and Devices, Guilin University of Technology, Guilin 541004, China; 2005032@glut.edu.cn

**Keywords:** SiO_x_ anode, lithium-ion battery, sandwich, pseudo-capacitance

## Abstract

SiO_x_ is considered as a promising anode for next-generation Li-ions batteries (LIBs) due to its high theoretical capacity; however, mechanical damage originated from volumetric variation during cycles, low intrinsic conductivity, and the complicated or toxic fabrication approaches critically hampered its practical application. Herein, a green, inexpensive, and scalable strategy was employed to fabricate NG/SiO_x_/NG (N-doped reduced graphene oxide) homogenous hybrids via a freeze-drying combined thermal decomposition method. The stable sandwich structure provided open channels for ion diffusion and relieved the mechanical stress originated from volumetric variation. The homogenous hybrids guaranteed the uniform and agglomeration-free distribution of SiO_x_ into conductive substrate, which efficiently improved the electric conductivity of the electrodes, favoring the fast electrochemical kinetics and further relieving the volumetric variation during lithiation/delithiation. N doping modulated the disproportionation reaction of SiO_x_ into Si and created more defects for ion storage, resulting in a high specific capacity. Deservedly, the prepared electrode exhibited a high specific capacity of 545 mAh g^−1^ at 2 A g^−1^, a high areal capacity of 2.06 mAh cm^−2^ after 450 cycles at 1.5 mA cm^−2^ in half-cell and tolerable lithium storage performance in full-cell. The green, scalable synthesis strategy and prominent electrochemical performance made the NG/SiO_x_/NG electrode one of the most promising practicable anodes for LIBs.

## 1. Introduction

Lithium-ion batteries (LIBs) have attracted extensive attention in the energy storage field, owing to their high energy density, long lifespan, and low self-discharge properties [1,2,3,4,5]. However, present LIB systems based on carbon as anode and lithium metal oxides as cathode with low energy densities cannot satisfy the ever-growing energy demands for electronic devices [6,7,8,9]. Recently, Si and SiO_x_ (0 < x ≤ 2) have caused widespread concern as promising alternative materials to traditional graphite anodes (with a limited specific capacity of 372 mAh g^−1^), due to their high theoretical capacities, relatively low redox potential vs. Li/Li^+^ and natural abundance features [8,10,11,12,13]. Meanwhile large volumetric variation over repeated delithiation/lithiation processes led to the particle fracture, the formation of unstable SEI (solid electrolyte interphase), loss contact between current collector and electrode materials, which contributed to its fast capacity decay; low ionic and electronic conductivity also restricted the rate capability [3,10,14,15]. For SiO_x_, the formation of Li_2_O and Li_4_SiO_4_ upon initial lithiation/delithiation process (Equations (1) and (2)) could act as the buffer layers to alleviate the volumetric variation of the in situ generated Si (Equation (1)), resulting in better cyclic performance of SiO_x_ than Si-based anodes [3,8,16,17]. Nonetheless, inevitable volumetric variation during the lithiation/delithiation process and intrinsic poor conductivity restricted the rate and cyclic performance of SiO_x_-based anodes [16,18,19].

SiO_x_ + 2xLi^+^ + 2xe^−^ → Si + xLi_2_O (1)

SiO_x_ + xLi^+^ + xe^−^ → (1 − x/4)Si + (x/4)Li_4_SiO_4_ (2)

Si + zLi^+^ + ze^−^ → Li_z_Si (3)

To overcome these obstacles, various strategies have been proposed to improve the conductivity and mitigate the volumetric variation of SiO_x_-based anodes. Constructing protective networks with conductive carbon, metals, polymers, MXene or other active materials can improve the conductivity, release the mechanical reaction stress and tolerate the volumetric variation of SiO_x_, thus efficiently prolonging the cyclic lifespan [2,3,8,16,20]. Designing hollow, york-shell, porous, and nanotube structures via etching or template strategies could provide void accommodation for volumetric variation and release mechanical stress, which could also enhance the electrochemical performance [21,22]. Nonetheless, most of the above strategies required complicated preparation processes or involved toxic chemical reagents, and the fabrication of SiO_x_-based anodes via simple, scalable, and green approaches is still imperative for its commercial application [14,21,23].

Integration of SiO_x_ with rGO (reduced graphene oxide) via physical mixing or sandwiching approaches attracted much attention because of the mild operation conditions [7,8,19]. The sandwich structure could provide stable frameworks and open channels for ion diffusion and electron transfer, thus sandwich hybrid electrodes usually exhibited superior electrochemical performance [10,24,25,26,27]. In addition, heteroatom-doped carbonaceous materials with additional active sites for ion storage and unique properties for electron transfer were proven as promising electrodes or substrates in energy storage devices [28,29,30]. However, fabricating homogenous SiO_x_/rGO without any agglomeration and unfavorable exposure of SiO_x_ or rGO is still a conundrum [19,29,31].

With the above in mind, we reported a green and scalable fabrication strategy to prepare SiO_x_/NG (N-doped rGO) homogenous hybrids via a freeze-drying combined thermal decomposition route. Characterization results indicated that SiO_x_ were encapsulated into rGO to form a sandwich like NG/SiO_x_/NG without any agglomeration and unfavorable exposure of SiO_x_ or rGO. The homogenous sandwich structure provided stable networks and open channels for electron transfer and ion diffusion, which could efficiently relieve the mechanical strain induced by volumetric variation of SiO_x_ during lithiation/delithiation; coated carbon and rGO improved the electronic conductivity, which favored the low polarization and fast electrochemical kinetic; N doping would create more defects, which provided additional ion storage sites and induced a pseudo-capacitance behavior. Consequently, the NG/SiO_x_/NG homogenous hybrids showed impressive electrochemical performance in half-cell and full-cell devices. The eco-friendly, scalable fabrication strategy, and prominent electrochemical performance made NG/SiO_x_/NG to be an ideal anode material for LIBs, and the eco-friendly, scalable fabrication strategy could be expanded to the preparation of other anode materials (Si, Sn, Ge etc.) with high energy densities.

## 2. Experimental Section

### 2.1. Material Preparation

Preparation of NG/SiO_x_/NG: Commercial SiO_x_ (Yuexing, Luoyang, China) was ball milled at a rotation speed of 600 rpm and dispersed into ethanol (Sinopharm Chemical Reagent Co., Ltd., Shanghai, China). Graphene oxide (GO, Sinopharm Chemical Reagent Co., Ltd., Shanghai, China) dispersion was prepared by the conventional modified Hummers method [30,32,33]. 30 mL GO dispersion (0.006 g mL^−1^), 6.3 mL SiO_x_ dispersion (0.028 g mL^−1^), and 0.95 g urea (Sinopharm Chemical Reagent Co., Ltd., Shanghai, China) were mixed together and ultrasonically dispersed to obtain a stable suspension. Finally, the suspension was freeze dried and calcined at 650 °C for 4 h with a heating rate of 2 °C min^−1^ in Ar. The obtained sample was labeled as NG/SiO_x_/NG.

Preparation of G/SiO_x_/G: the preparation process of G/SiO_x_/G was similar to that of NG/SiO_x_/NG except that no urea was added into the suspension.

### 2.2. Material Characterizations

Powder X-ray diffraction (PXRD) patterns were captured on a Bruker D8 (Bruker AXS Co., Karlsruhe, Germany) X-ray diffractometer using Cu Kα radiation. Thermogravimetric analysis (TGA) was recorded on a TG/DTA 6300 thermogravimetric analyzer (Perkin-Elmer Inc., Boston, MA, USA). N_2_ adsorption/desorption isotherms were conducted by using a Tristar 3000 micrometric apparatus (Micromeritics Corporate, Norcross, GA, USA) at −196 °C. Raman spectra were recorded on an HR-800 μ-Raman system (HORIBA Jobin Yvon, Paris, France) using an Ar laser (514.5 nm). Transmission electron microscopy (TEM), high-resolution TEM (HRTEM), and energy-dispersive X-ray spectroscopy (EDX) were captured on a JEOL JEM-2100F field-emission TEM (JEOL Ltd., Tokyo, Japan). Scanning electron microscopy (SEM) images were obtained on a Zeiss Sigma-500 field-emission SEM (Carl Zeiss AG Oberkochen, Germany). X-ray photoelectron spectroscopy (XPS) spectra were employed to analyze the chemical state of the samples using a PHI-1600 photoelectron spectrometer (ULVAC-PHI Inc., Tokyo, Japan) with Al-Kα X-ray source.

### 2.3. Electrochemical Characterization

CR2032 coin-cells were assembled in an Ar-filled glove box (Omni-LAB, Vacuum Atmospheres Company, Lawndale, CA, USA) to survey the electrochemical performance of the prepared composites, metallic Li (Shenzhen Teesky Technology Co., Ltd., Shenzhen, China) or commercial LiFePO_4_ (Shenzhen Teesky Technology Co., Ltd., Shenzhen, China) were used as the counter electrode. A slurry consisting of 70 wt.% active composite, 20 wt.% conductive carbon black (Shenzhen Teesky Technology Co., Ltd., Shenzhen, China) and 10 wt.% polyvinylidene fluoride (PVDF, Shenzhen Teesky Technology Co., Ltd., Shenzhen, China) with N-methylpyrrolidone (NMP, Energy Chemical, Shanghai, China) as solvent were coated on Cu/Al (Shenzhen Teesky Technology Co., Ltd., Shenzhen, China) foil and then dried at 120 °C for 24 h. 1 mol L^−1^ LiPF_6_ in a mixture of ethylene carbonate, dimethyl carbonate (1/1 *v*/*v*) and 5 wt.% fluoroethylene carbonate (FEC) were used as the electrolyte (Shenzhen Teesky Technology Co., Ltd., Shenzhen, China). The mass loading of the anode composites on Cu foil was ~1 mg cm^−2^, and the capacity of anode material in half-cells was calculated based on the weight of anode composites. The mass ratio of negative and positive materials in full-cells was ~0.12, and the mass capacity of full-cells were calculated based on the weight of LiFePO_4_. The NG/SiO_x_/NG anode was activated for 10 cycles in half-cells before assembling NG/SiO_x_/NG//LiFePO_4_ full-cell. Galvanostatic charge/discharge (GCD) tests were conducted using a Neware (CT-3008W, Shenzhen Neware Technology Limited, Shenzhen, China) instrument. The electrochemical impedance spectroscopy (EIS) and cyclic voltammetry (CV) curves were captured on a Parstat 4000+ electrochemical workstation (Princeton Applied Research, Princeton, United States). The voltage windows were set as 3–0.05 V and 2.8–4.2 V for half- and full-cells, respectively.

## 3. Results and Discussion

**Figure 1:**
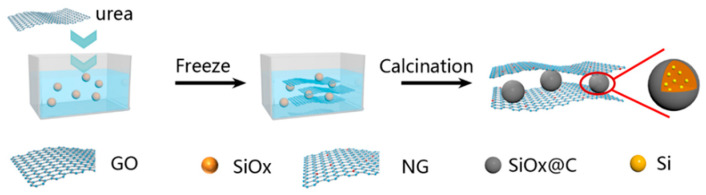
Schematic diagram for the fabrication process of the sandwich-like NG/SiO_x_/NG composites.

NG/SiO_x_/NG composite was prepared by a nontoxic, simple, and scalable freeze-drying combined thermal decomposition strategy, as illustrated in Figure 1. After mechanical ball-milling, commercial SiO_x_ was crushed and dispersed into ethanol. Subsequently, SiO_x_ suspension was mixed with GO-urea homogeneous solution uniformly. Under ultrasound treatment, SiO_x_ particles were bundled into GO and surrounded by urea. The freeze-drying strategy maintained the sandwich structure during the drying process. During the thermal treatment, GO was reduced to rGO, partial SiO_x_ was decomposed into Si and SiO_2_, urea was decomposed into N-doped carbon and partial N was doped into rGO. No poisonous chemicals and hydrothermal process were used during the fabrication process of sandwich-like NG/SiO_x_/NG composite, in other words, the fabrication process is green and scalable.

**Figure 2:**
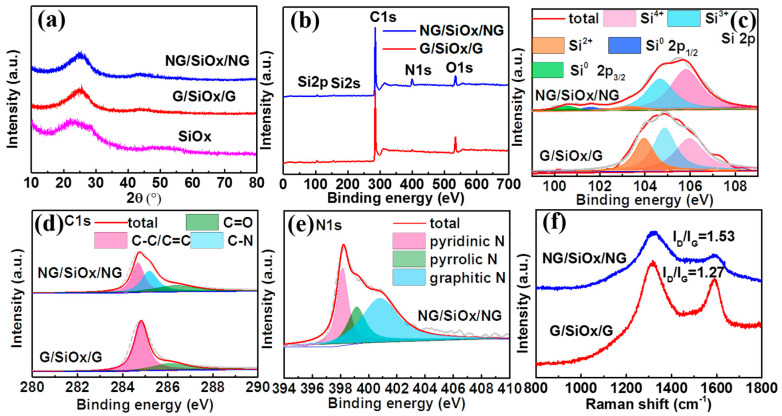
Structural characterizations of G/SiO_x_/G and NG/SiO_x_/NG: (**a**) XRD patterns; (**b**) total XPS spectra; (**c**) Si 2p, (**d**) C1s and (**e**) N 1s XPS spectra; (**f**) Raman spectra.

The crystal structures are determined by PXRD and the results are demonstrated in Figure 2a and Figure S1. For GO, a typical diffraction peak located below 15° could be observed in Figure S1; while after calcination in Ar at 650 °C, the diffraction peak below 15° disappeared and a new diffraction peak located at 26° appeared, which should be attributed to the removal of oxygen containing functional groups and the exfoliation of multiple layers during the thermal reduction of GO [33,34,35]. The variation of PXRD patterns for GO after calcination confirmed the thermal reduction of GO into rGO under Ar. For SiO_x_, two broad diffraction peaks located around 25° and 45° can be observed (Figure 2a), indicating the amorphous or low crystallinity structure of SiO_x_ in the samples. No diffraction peak corresponding to GO (below 15°) can be detected in Figure 2a, meaning that GO was successfully reduced to rGO in G/SiO_x_/G and NG/SiO_x_/NG [19,33]. Therefore, two broad diffraction peaks located around 25° and 45° in G/SiO_x_/G and NG/SiO_x_/NG could be assigned to the superimposed diffraction peaks of SiO_x_ and rGO. Generally, SiO would be decomposed into Si and SiO_2_ under heat treatment [14,36,37]. In this work, no diffraction peaks assigned to Si can be observed, which can be attributed to the small crystalline size of Si. XRD results verify the successful fabrication of SiO_x_/G composites.

XPS spectra were conducted to investigate the chemical state of Si, O, C, and N in the samples and the results are displayed in Figure 2b–f. The very weak signals of Si 2p and Si 2s in Figure 2b can be attributed to the covering of rGO on the surface of SiO_x_. Three peaks centered at 105.8, 104.8, and 103.9 eV are detected for Si 2p (Figure 2c), indicating the co-existing of Si^4+^, Si^3+^, and Si^2+^ in the two samples [19,33,36,38,39]. Different from G/SiO_x_/G, new binding energies located at 100.6 and 101.6 eV assigned to Si^0^ 2p_3/2_ and Si^0^ 2p_1/2_ can be observed in NG/SiO_x_/NG; while the peak corresponding to Si^2+^ became lower and Si^4+^ became stronger, indicating that partial SiO was decomposed into Si and SiO_2_ during the thermal treatment [14,19,31,40]. In other words, the gas released during the decomposition of urea or N doping can promote the disproportionation of SiO. According to the valence of silicon, the atomic ratios of O/Si were 1.55 and 1.68 in G/SiO_x_/G and NG/SiO_x_/NG, respectively (Table 1). Two Lorentzian peaks of C1s located at 284.7 and 286.4 eV represented the C-C/C=C and C=O bonds, respectively; and the additional peak centered at 285.2 eV in NG/SiO_x_/NG was assigned to C-N bond (Figure 2d) [33,39,41,42,43]. The N 1s spectrum in Figure 2e exhibited three typical peaks at 398.2, 399.2, and 400.8 eV, which could be attributed to pyridinic N, pyrrolic N, and graphitic N, respectively [38,39,44,45,46]. The XPS results indicated that N doping facilitated the disproportionation of SiO, resulting in the formation of abundant SiO_2_ shells and high dispersive Si cores in SiO_x_/NG composite.

Raman spectra of G/SiO_x_/G and NG/SiO_x_/NG in Figure 2f displayed two typical broadened bands at 1315.8 and 1587.4 cm^−1^, which are assigned to the disordered band (D-band) and graphite band (G-band) of carbonaceous materials, respectively [3,19,44]. The D-band to G-band intensity ratios (I_D_/I_G_) were calculated to be 1.53 and 1.27 for NG/SiO_x_/NG and G/SiO_x_/G, indicating that the main component of carbon in both samples were amorphous; the higher I_D_/I_G_ in NG/SiO_x_/NG implied that more defects existed in N-doped rGO, which is beneficial to electronic conductivity and the ion storage of NG/SiO_x_/NG composite [19,44]. TGA results in Figure S2a showed that the carbon content in NG/SiO_x_/NG and G/SiO_x_/G were 21.7% and 16.7%, respectively. In addition, the surface area increased from 4 to 34 m^2^ g^−1^ after N doping (Figure S2b), the higher surface area favored the electrolyte infiltration and the electrochemical kinetics in NG/SiO_x_/NG [43,47].

**Figure 3:**
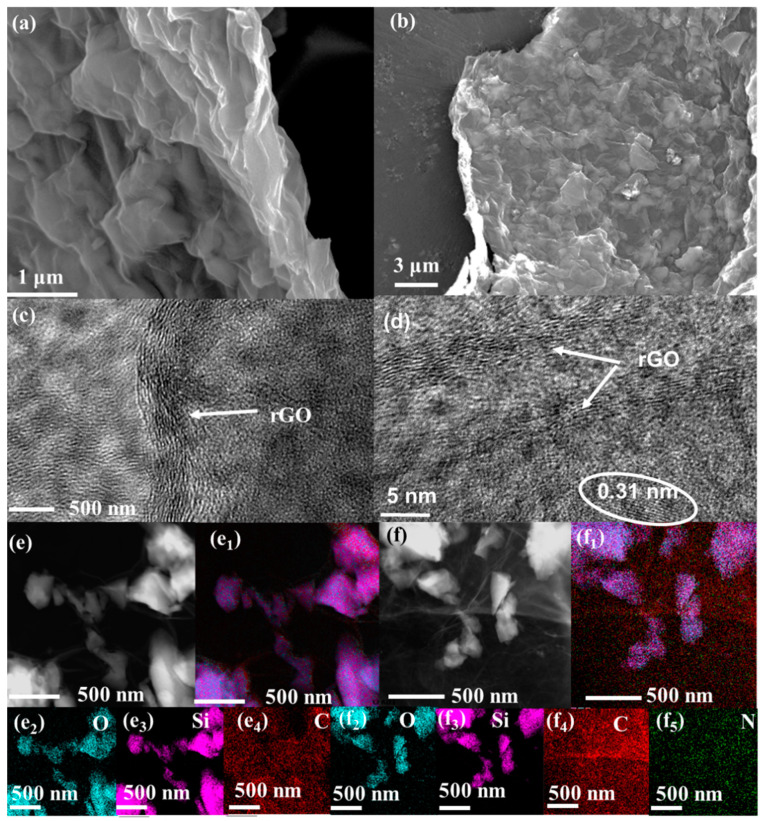
Morphological characterizations: (**a**) SEM and (**c**) TEM pictures of G/SiO_x_/G; (**b**) SEM and (**d**) TEM pictures of NG/SiO_x_/NG; TEM and the corresponding elemental mappings of (**e**) G/SiO_x_/G and (**f**) NG/SiO_x_/NG.

The NG/SiO_x_/NG and G/SiO_x_/G composites both exhibited 2D nanosheet flake morphologies, which is the typical morphology of rGO (Figure 3a,b and Figure S3a,b). The enlarged pictures in Figure 3a,b showed that internal particles popped up many protrusions on the surface of rGO, indicating that SiO_x_ was stacked and encapsulated into rGO nanosheets. More importantly, no agglomeration and unfavorable exposure of SiO_x_ or rGO could be seen in Figure 3a,b and Figure S3a,b, indicating the formation of sandwich-like SiO_x_/G homogeneous hybrids. TEM results in Figure S3c,d further proved the formation of sandwich-like structure of the prepared composites. In Figure 3d, lattice fringes with a *d*-spacing of 0.31 nm corresponding to Si (111) can be observed, indicating the partial decomposition of SiO into Si in NG/SiO_x_/NG, which is consistent with the XPS results. In accordance with the SEM and TEM results, the elements distributions in Figure 3e,f also indicated that the SiO_x_ particles were trapped and encompassed by the conductive carbon tightly in G/SiO_x_/G and NG/SiO_x_/NG. The uniform distribution of N and C in Figure 3f further confirmed the successful doping of N into carbon in NG/SiO_x_/NG, agreeing with the XPS results. Morphological characterizations verified the formation of sandwich-like structure of SiO_x_/G and SiO_x_/NG homogeneous hybrids; in other words, NG/SiO_x_/NG and G/SiO_x_/G homogeneous hybrids were successfully fabricated by the green, simple, and scalable method.

**Figure 4:**
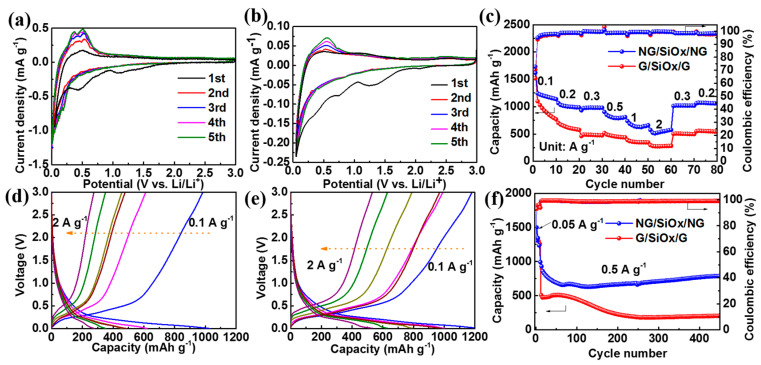
Electrochemical performance in half-cells: CV curves of (**a**) G/SiO_x_/G and (**b**) NG/SiO_x_/NG at 0.1 mV s^−1^; (**c**) rate performance; GCD curves of (**d**) G/SiO_x_/G and (**e**) NG/SiO_x_/NG at different current densities; (**f**) cycling performance at 0.5 A g^−1^.

The lithium storage performance of G/SiO_x_/G and NG/SiO_x_/NG were investigated in half-cells, firstly. The CV curves at 0.1 mV s^−1^ in Figure 4a,b both exhibited broad irreversible peaks at 0.3–1.2 V in the first cathodic scan, attributing to the formation of SEI, the decomposition of electrolyte/FEC, and the lithiation of SiO_x_ into Si and Li_2_O/Li_4_SiO_4_ (Equations (1) and (2)) [36,44,48,49]. And the distinct cathodic peak below 0.25 V can be assigned to the lithiation of Si into Li_x_Si alloys (Equation (3)) [3,21,36,50]. The charging branch showed broad peaks at 0.3–0.6 V, which can be attributed to the multi-step de-alloying processes of Li_x_Si alloys [10,17,21,36,51]. The CV areas increased in the following cycles, which was associated with the activation of the electrode material and the improved contact between SiO_x_ particles and coated carbonaceous [17,21,36]. The GCD curves of SiO_x_, G/SiO_x_/G, and NG/SiO_x_/NG at 0.1 A g^−1^ are demonstrated in Figure S4. Bulk SiO_x_ delivered a high initial discharge capacity of 2718.3 mAh g^−1^ with a low coulombic efficiency of 49.6%. The G/SiO_x_/G electrode exhibited initial discharge and charge capacities of 1678.5 mAh g^−1^ and 1070.8 mAh g^−1^ with a coulombic efficiency of 63.8%. N doping would create more defects and more active sites for ion adsorption, deservedly, the NG/SiO_x_/NG electrode delivered a higher initial charge capacity of 1182.3 mAh g^−1^ with a higher coulombic efficiency of 66.6%. The overlapped GCD curves in the following cycles indicated the high electrochemical reversibility of NG/SiO_x_/NG [1,23,43]. The improved coulombic efficiency from 49.6% for SiO_x_ to 66.6% for NG/SiO_x_/NG and the high reversibility verified the NG/SiO_x_/NG electrode to be a promising anode for LIBs (Table S1).

The rate performance and cyclic performance in Figure 4c–f further verified the superior electrochemical performance of NG/SiO_x_/NG. The capacities of the G/SiO_x_/G electrode decreased quickly with increasing densities (Figure 4c,d) and, typically, the average capacities were 928.4, 619.6, 479.2, 463.8, 362.1, 276.5 mAh g^−1^ at 0.1, 0.2, 0.3, 0.5, 1, and 2 A g^−1^, respectively; when the current density recovered to 0.2 A g^−1^ from 2 A g^−1^, the capacity was 561.1 mAh g^−1^. After 100 cycles at 0.5 A g^−1^, the specific capacity of G/SiO_x_/G decreased dramatically, and only a stable capacity of 212.5 mAh g^−1^ was retained after 450 cycles (Figure 4f). In contrast, the NG/SiO_x_/NG demonstrated higher capacities of 1197.6, 1002.3, 978.9, 803.3, 643.1, and 545.8 mAh g^−1^ at above current densities; when the current density recovered to 0.2 A g^−1^ from 2 A g^−1^, a high capacity of 1072.6 mAh g^−1^ was obtained, indicating the high reversibility of the NG/SiO_x_/NG electrode (Figure 4c,e). Moreover, after 450 cycles at 0.5 A g^−1^, the NG/SiO_x_/NG electrode maintained a high reversible capacity of 798.9 mAh g^−1^ with a loading amount of 1 mg cm^−2^ (Figure 4f) and a comparable reversible capacity of 696.9 mAh g^−1^ with a higher loading amount of 3 mg cm^−2^ (Figure S5). The comparison of the electrochemical performance between the state-of-the-art SiO_x_ electrodes and the NG/SiO_x_/NG electrode also verified the superior lithium storage properties of NG/SiO_x_/NG (Table S1).

**Figure 5:**
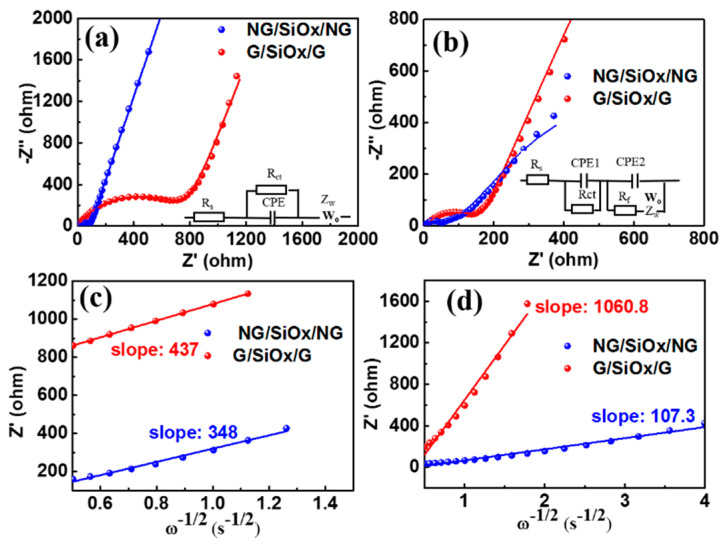
Electrochemical kinetics of G/SiO_x_/G and NG/SiO_x_/NG electrodes: EIS spectra (**a**) before and (**b**) after 450 cycles; the corresponding Z′ − ω^−1/2^ plots in the low-frequency region of electrochemical impedance (**c**) before and (**d**) after 450 cycles.

**Figure 6:**
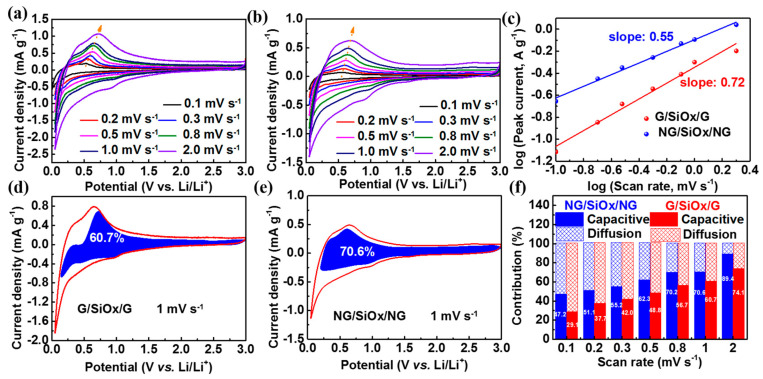
Electrochemical charge storage mechanism of G/SiO_x_/G and NG/SiO_x_/NG in half-cells: CV curves of (**a**) G/SiO_x_/G and (**b**) NG/SiO_x_/NG at different scan rates; (**c**) log (*i*) − log (*v*) plots to determine *b* values; pseudo-capacitance contribution of (**d**) G/SiO_x_/G and (**e**) NG/SiO_x_/NG at 1 mV s^−1^; (**f**) individual contribution of pseudo-capacitive and diffusion-controlled behaviors at different scan rates.

To understand the dynamic electrochemical properties of the NG/SiO_x_/NG electrode, the electrochemical kinetics and charge storage mechanism were investigated and demonstrated in Figure 5 and Figure 6. Semicircles in the high-frequency region corresponding to the SEI resistance (R_f_) and charge transfer resistance (R_ct_) could be observed in the Nyquist diagrams of the G/SiO_x_/G and NG/SiO_x_/NG electrodes after 450 cycles (Figure 5b) [3,52,53]. In the corresponding fresh cells, no semicircles assigned to R_f_ were detected since the absence of SEI (Figure 5a) [52]. Smaller R_f_ and R_ct_ values (Table S2) of the Li//NG/SiO_x_/NG cell than those of the Li//G/SiO_x_/G cell indicated the enhanced conductivity of the NG/SiO_x_/NG composite by N doping, which resulted in its fast electrode kinetics [3,10,17,52]. Slope lines in the low-frequency region are ascribed to Warburg impedance (Z_w_), which is associated with Li^+^ diffusion in bulk electrode materials [18,47,54]. And the lithium ion diffusion coefficient (D_Li+_) could be calculated with Equation (4) through linear fitting between Z’ and ω^−1/2^ (ω = 2πf) in low frequencies of Nyquist diagrams [1,3]. In Equation (4), R, T, A, n, F, and C are the gas constant, absolute temperature, surface area of the electrode, number of transferred electrons per molecule in the material, Faraday’s constant and concentration of Li^+^, respectively; while σ represented the Warburg coefficients and could be determined by the slopes of Z′ − ω^−1/2^ plots (Figure 5c,d) [3,29,52]. According to Equation (4), for a given electrode, the σ^−2^ value is proportional to D_Li+_ while all the other parameters are constants [1,18,55]. The much smaller σ^−2^ values (Table S2) of the NG/SiO_x_/NG electrode indicated the faster diffusion of the Li^+^ in NG/SiO_x_/NG electrode [53,54,56]. The lower electrode impedance and faster diffusion of Li^+^ contributed to the enhanced electrochemical capacity and rate capability of the NG/SiO_x_/NG electrode.

D_Li+_ = R^2^T^2^/(2A^2^n^4^F^4^σ^2^C^2^) (4)

To have thorough insights into the underlying mechanism of the superior electrochemical performance of the NG/SiO_x_/NG electrode, CV curves at scan rates ranging from 0.1 to 2 mV s^−1^ were measured to estimate the charge storage process. The anodic and cathodic peaks exhibited similar shapes with smaller potential shifts with increasing scan rates (Figure 6a,b) in the NG/SiO_x_/NG electrode, indicating its faster reaction kinetic and lower polarization during the lithiation/delithiation process [1,57]. To date, two charge storage mechanisms of the diffusion-controlled intercalation/alloying/conversion process and the capacitance-controlled pseudocapacitive process are involved in various electrode materials for LIBs [23,43,58]. The power-law relationship between the measured current (*i*) and sweep rates (*v*) is generally expressed as Equation (5), where *a* and *b* are adjustable parameters [1,43,57,59]. The *b* values could be determined by slopes of log (*v*) − log (*i*) plots, and two typical *b* values of 1 and 0.5 indicated that the electrode was dominated by capacitive behaviors and diffusive behaviors, respectively [45,54,57]. The calculated *b* values at low potentials are 0.72 and 0.55 in the NG/SiO_x_/NG and G/SiO_x_/G electrodes (Figure 6c), indicating the hybrid contributions of diffusion-controlled behavior and capacitance-controlled behavior to the total capacity; and the larger *b* value for the NG/SiO_x_/NG electrode suggested that the charge storage was mainly dominated by surface capacitance-controlled behavior, which would result in a faster electrode kinetic [23,29,52].

*i* = *a v^b^* (5)

*i*(*V*) = k_1_*v* + k_2_*v*^1/2^ (6)

The quantitative contribution of surface capacitance-controlled behavior and diffusion-controlled behavior could be calculated by separating the total current response at a fixed potential according to Equation (6), where *i* (*V*), k_1_*v* and k_2_*v*^1/2^ represented the total current, capacitance contributed current and diffusion contributed current, respectively [29,45,52]. According to Equation (6), k_1_ values at different voltages could be determined by the slopes of *i* (*V*)/*v*^1/2^ − *v*^1/2^ plots, and then k_1_*v* − V plots at different scan rates could be obtained. The pseudocapacitive contribution ratios at different scan rates could be quantified by the area ratios of k_1_*v* − V plots (which were filled with a blue color in Figure 6d,e) and CV curves. The calculated capacity contribution from the diffusion-controlled behavior and surface-capacitive behavior (blue area) at 1 mV s^−1^ are demonstrated in Figure 6d,e. The surface-capacitive behavior contributed 60.7% and 70.6% to the total storage capacity at 1 mV s^−1^ for the G/SiO_x_/G and NG/SiO_x_/NG electrodes, respectively; and the capacitive contribution ratios to total stored charge increased with increasing scan rates, implying that capacitive charge-storage mechanism mainly dominated the total charge storage; especially for the NG/SiO_x_/NG electrode, which possessed higher capacitive contribution ratios at all scan rates [1,45,57]. The above electrochemical charge storage mechanism analysis further revealed the fast electrochemical kinetics and explained the enhanced rate capability of the NG/SiO_x_/NG electrode exhibited in Figure 4. The probable reasons might be that N-doped rGO with high conductivity provided an affluent high-speed path to lowering the electrode impedance for ion diffusion.

The morphological stability of the G/SiO_x_/G and NG/SiO_x_/NG electrodes after 450 cycles were carried out to deeply understand the enhanced electrochemical performance by N doping into G/SiO_x_/G. The thickness of the cycled NG/SiO_x_/NG only increased from 10.7 μm to 11.6 μm with a 17.7% volume expansion; while the G/SiO_x_/G electrode displayed a larger swelling rate of 146.5% and a peel-off phenomenon of the electrode materials from Cu foil, resulting in loss of the electrical contact and the fast capacity fading during cycling (Figure S6a–d) [10,20]. Furthermore, after 450 cycles, the sandwich structure of NG/SiO_x_/NG maintained, while that of G/SiO_x_/G obviously collapsed, the stable sandwich structure relieved the mechanical stress caused by volumetric variation during the lithiation/delithiation process and contributed partially to the stable electrochemical performance of the NG/SiO_x_/NG nanocomposites (Figure S6e–h) [10,20,47].

**Figure 7:**
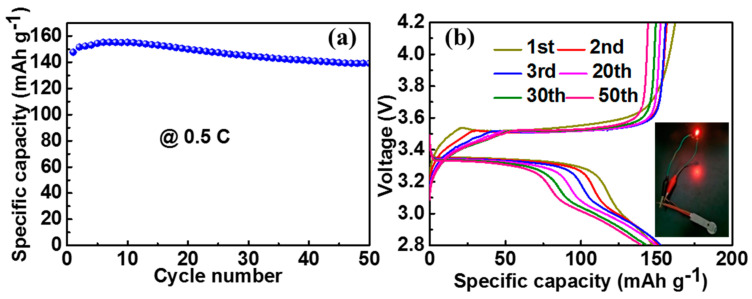
Electrochemical performance in full-cells: (**a**) cyclic performance and (**b**) GCD curves of NG/SiO_x_/NG//LiFeO_4_ at 0.5 C; inset in b showed the alight LED bulb by the NG/SiO_x_/NG//LiFePO_4_ full-cell.

To demonstrate the potential application of NG/SiO_x_/NG, full-cells using NG/SiO_x_/NG as anode and commercial LiFePO_4_ as cathode were assembled. The LiFePO_4_ cathode delivered high discharge capacities of 147.6 mAh g^−1^, 150 mAh g^−1^, and 140 mAh g^−1^ at the 1st, 20th, and 50th cycles at 0.5 C (1 C = 170 mA g^−1^), respectively (Figure 7a). More importantly, the NG/SiO_x_/NG//LiFePO_4_ full-cell outputted a stable voltage platform at 3.3 V, and the red-light-emitting diode bulb could be easily powered by the prepared NG/SiO_x_/NG//LiFePO_4_ full-cell (Figure 7b). The results strongly indicated that the NG/SiO_x_/NG electrode was a potential practical anode for LIBs.

## 4. Conclusions

Sandwich-like NG/SiO_x_/NG was successfully fabricated through a freeze-drying combined thermal decomposition strategy. When used as the anodes for LIBs, a high specific capacity of 545 mAh g^−1^ at 2 A g^−1^, stable capacity of 799 mAh g^−1^ after 450 cycles at 0.5 A g^−1^ and high areal capacity of 2.06 mAh cm^−2^ after 450 cycles at 1.5 mA cm^−2^ were obtained in half-cells; NG/SiO_x_/NG//LiFePO_4_ full-cells exhibited a high output voltage of 3.3 V and a tolerable specific capacity. The superior electrochemical performance can be attributed to the following features of the NG/SiO_x_/NG electrode: (i) the uniform and agglomeration-free package of SiO_x_ into rGO efficiently improved the electric conductivity of the electrode, lowering the electrode impedance and favoring the fast electrochemical kinetics; (ii) the stable sandwich structure relieved the mechanical stress caused by volumetric variation during the lithiation/delithiation process, resulting in the prolonged cyclic life; (iii) nano Si produced from the disproportionation reaction of SiO_x_ and defects originated from N doping created more active sites for ion storage, resulting in the high specific capacity; (iv) N doping induced a strong capacitive contribution to ion storage, improving the electrochemical kinetics. The green, scalable fabrication strategy and superior electrochemical performance indicated that the NG/SiO_x_/NG electrode should be a promising practicable electrode for LIBs. In addition, the simple and universal strategy can also be expanded to the fabrication of other electrodes (Si, Sn and Ge etc.) with high densities.

## Figures and Tables

**Table 1:** Molar percentages of Si^4+^, Si^3+^, Si^2+^, Si^0^ and molar ratios of O/Si in the composites calculated from XPS results.

Sample	Si^4+^	Si^3+^	Si^2+^	Si^0^	O/Si Ratio
G/SiO_x_/G	36.4%	37.9%	25.7%	0%	1.55
NG/SiO_x_/NG	53.2%	38.1%	4.6%	4.1%	1.68

## Data Availability

The data supporting the findings of this study are contained within the article and supplementary materials. And the initial data are available from the first author upon reasonable request via email.

## Supplementary Materials

The following are available online at https://www.mdpi.com/article/10.3390/nano11092366/s1, Figure S1: PXRD patterns of GO obtained from thermal drying of GO dispersion and rGO from calcination of GO in Ar at 650 °C. Figure S2: TGA curves and N_2_ adsorption-desorption isotherms of G/SiO_x_/G and NG/SiO_x_/NG. Figure S3: SEM and TEM pictures of G/SiO_x_/G and NG/SiO_x_/NG. Figure S4: GCD curves of SiO_x_, G/SiO_x_/G and NG/SiO_x_/NG at 0.1 A g^−1^. Figure S5: Cyclic performance of NG/SiO_x_/NG electrode with loading amount of 1 mg cm^−2^, 3 mg cm^−2^ and 3 mg cm^−2^ (8:1:1); where 8:1:1 represented that the electrode were prepared with a mass ratio of active material:conductive carbon:PVDF = 8:1:1. Figure S6: Morphological stability of G/SiO_x_/G and NG/SiO_x_/NG electrodes: the initial electrode disks for NG/SiO_x_/NG and G/SiOx/G; the electrode disks for NG/SiO_x_/NG and G/SiO_x_/G, morphologies for NG/SiO_x_/NG and G/SiO_x_/G after 450 cycles at 0.5 A g^−1^. Table S1: Rate and cyclic performance of the state-of-the-art SiO_x_ electrodes. Table S2: Impedance parameters determined from the EIS results of G/SiO_x_/G and NG/SiO_x_/NG before and after 450 cycles in half-cells.
