# Supplementary material for: Green and Scalable Fabrication of Sandwich-like NG/SiOx/NG Homogenous Hybrids for Superior Lithium-Ion Batteries
Source: Nanomaterials (Basel). 2021 Sep 11;11(9):2366. doi: 10.3390/nano11092366 (PMC8467742; doi:10.3390/nano11092366)
Supplement: Supplementary file 1 [file nanomaterials-11-02366-s001.zip › nanomaterials-1366787-supplementary.pdf]

nanomaterials

Supporting Information

Green and Scalable Fabrication of Sandwich-like NG/SiO_x/NG Homogenous Hybrids for Superior Lithium-Ion Batteries

Guilong Liu [1], Yilin Wei [1], Tiantian Li [1], Yingying Gu [1], Donglei Guo [1], Naiteng Wu [1], Aimiao Qin [2] and Xianming Liu [1,*]

[1] Key Laboratory of Function-Oriented Porous Materials of Henan Province, College of Chemistry and Chemical Engineering, Luoyang Normal University, Luoyang 471934, China; glliu@tju.edu.cn (G.L.); yilinwei27@163.com (Y.W.); Lt248861494@163.com (T.L.); gy17335979115@163.com (Y.G.); gdl0594@163.com (D.G.); wunaiteng@gmail.com (N.W.)

[2] Key Laboratory of New Processing Technology for Nonferrous Metal & Materials, Guangxi Key Laboratory of Optical and Electronic Materials and Devices, Guilin University of Technology, Guilin 541004, China; 2005032@glut.edu.cn

* Correspondence: lxm-nanoenergy@lynu.edu.cn

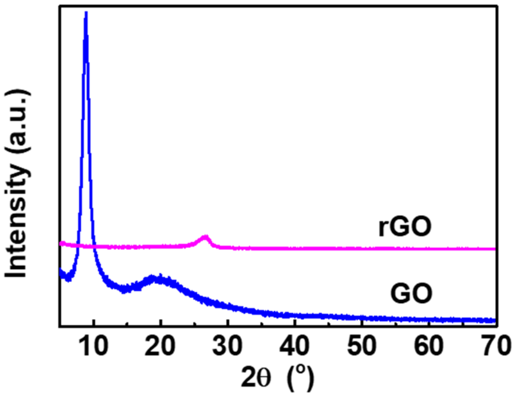

Figure S1. PXRD patterns of GO obtained from thermal drying of GO dispersion and rGO from calcination of GO in Ar at 650 °C.

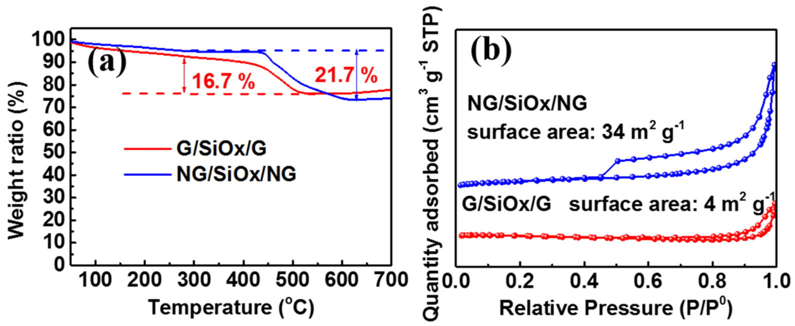

Figure S2. (**a**) TGA curves and (**b**) N_2 adsorption-desorption isotherms of G/SiO_x/G and NG/SiO_x/NG.

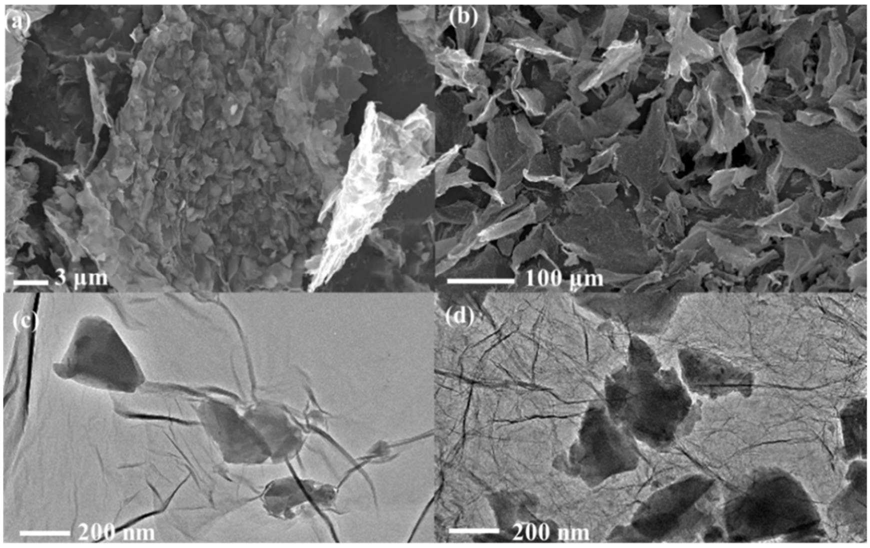

Figure S3. (**a**) SEM and (**c**) TEM pictures of G/SiO_x/G; (**b**) SEM and (**d**) TEM pictures of NG/SiO_x/NG.

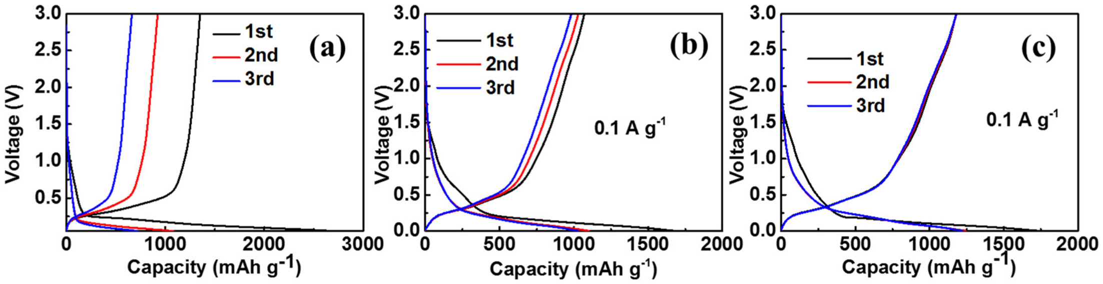

Figure S4. GCD curves of (**a**) SiO_x, (**b**) G/SiO_x/G and (**c**) NG/SiO_x/NG at 0.1 A g^{-1}.

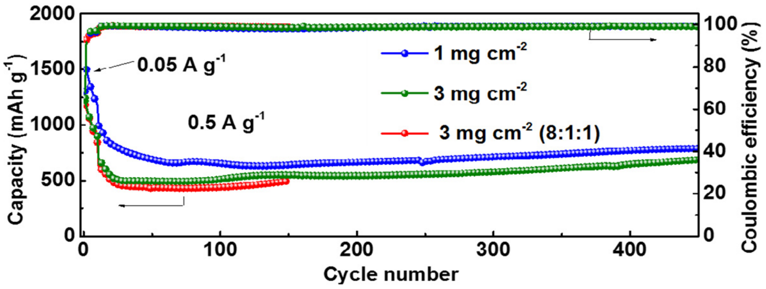

Figure S5. Cyclic performance of NG/SiO_x/NG electrode with loading amount of 1 mg cm^{-2}, 3 mg cm^{-2} and 3 mg cm^{-2} (8:1:1); where 8:1:1 represented that the electrode were prepared with a mass ratio of active material:conductive carbon:PVDF = 8:1:1.

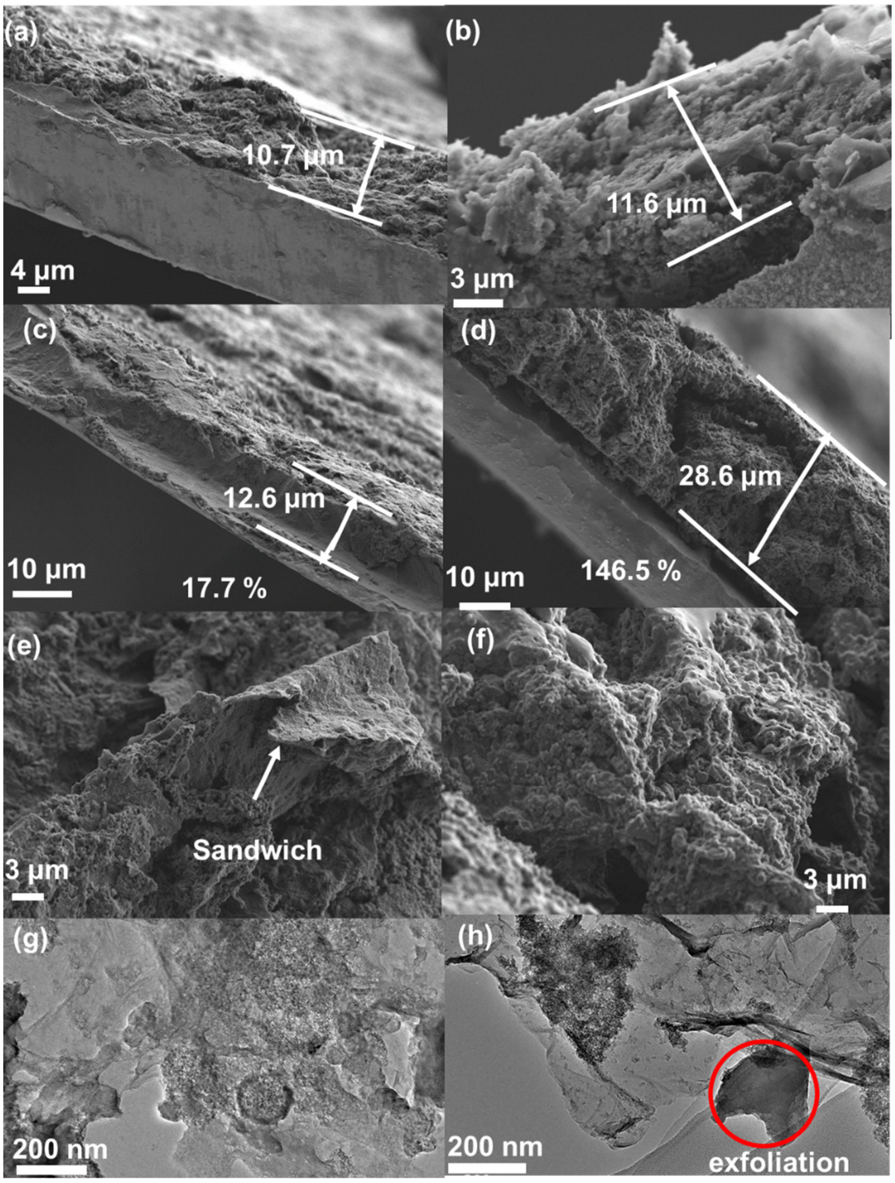

Figure S6. Morphological stability of G/SiO_x/G and NG/SiO_x/NG electrodes: the initial electrode disks for (**a**) NG/SiO_x/NG and (**b**) G/SiO_x/G; the electrode disks for (**c**) NG/SiO_x/NG and (**d**) G/SiO_x/G, morphologies for (**e**,**g**) NG/SiO_x/NG and (**f**,**h**) G/SiO_x/G after 450 cycles at 0.5 A g^{-1}.

Table S1. Rate and cyclic performance of the state-of-the-art SiO_x electrodes.

**Materials**	**SiO_x Content** [a]	**Electrode Composition** [b]	**Loading Amount** [c]	**O/Si Ratio**	**ICE** [d]	**Rate Performance**	**Capacity Retention** [e]	**Cycling Performance**	**Reference**
Si@SiO_x@$TiO_{2-\delta}$	~	60%	1.6		83.7%	310 mAh g^{-1} at 1 A g^{-1}	42.4% [f]	650 mAh g^{-1} after 200 cycles at 0.2 A g^{-1}	[1]
SiO_{2-x}/MXene	~76%	80%	2.58	-	71.0%	553 mAh g^{-1} at 2 A g^{-1}	65.8%	798 mAh g^{-1} after 100 cycles at 0.2 A g^{-1}	[2]
Si/SiO_x@NC	85.4%	70%	~0.65	-	~49%	161 mAh g^{-1} at 2 A g^{-1}	22.5%	503 mAh g^{-1} after 400 cycles at 0.5 A g^{-1}	[3]
$SiO_{1.47}$@C	54.5%	80%	-	1.47	67.2%	~550 mAh g^{-1} at 1.5 A g^{-1}	~61.1%	700 mAh g^{-1} after 300 cycles at 0.75 A g^{-1}	[4]
SiO_x/C-2	83.4%	70%	1−1.5	1.41	61.0%	532 mAh g^{-1} at 2 A g^{-1}	45.8%	872 mA h g^{-1} after 200 cycles at 0.5 A g^{-1}	[5]
SiO/1D-C/a-C	65.2%	75%	~1.2	-	70.2%	~300 mAh g^{-1} at 2 A g^{-1}	~25%	~1000 mAh g^{-1} after 120 cycles at 0.1 A g^{-1}	[6]
SiO_x@NC	44.6%	70%	1.0−1.5	1.75	59.1%	~450 mAh g^{-1} at 2 A g^{-1}	~	774 mAh g^{-1} after 100 cycles at 0.2 A g^{-1}	[7]

Si/SiO_x@CNF	11.3%	-	-	-	~	417 mAh g^{-1} at 2 A g^{-1}	31.1% [g]	600 mAh g^{-1} after 100 cycles at 3 A g^{-1}	[8]
SiO_x-TiO_2@RGO	58.1%	80%	-	-	51.1%	550 mAh g^{-1} at 2 A g^{-1}	~55%	470 mAh g^{-1} after 500 cycles at 0.5 A g^{-1}	[9]
HSiO_2@CN	57%	70%	-	-	<60%	490 mAh g^{-1} at 2 A g^{-1}	61.2%	810 mAh g^{-1} after 100 cycles at 0.2 A g^{-1}	[10]
SiO_2/C@SiO_2@C	72.1%	60%	~1	-	<64.2%	370 mAh g^{-1} at 1 A g^{-1}	61.2%	644 mAh g^{-1} after 200 cycles at 0.1 A g^{-1}	[11]
NG/SiO_x/NG	78.6%	70%	~1<br>~3	1.68	66.6%	545 mAh g^{-1} at 2 A g^{-1}	45.6%	799 mAh g^{-1} after 450 cycles at 0.5 A g^{-1}<br>697 mAh g^{-1} after 450 cycles at 0.5 A g^{-1}	This work

[a] Mass fraction of SiO_x in the composites; [b] Mass fraction of active materials in the slurry coated on copper; [c] Loading amount of electrode materials on Cu foil, the unit is mg cm^{-2}; [d] Initial coulombic efficiency; [e] The ratio of the capacity at 2 A g^{-1} and 0.1 A g^{-1}; [f] The ratio of the capacity at 1 A g^{-1} and 0.1 A g^{-1}; [g] The ratio of the capacity at 2 A g^{-1} and 0.3 A g^{-1}.

Table S2. Impedance parameters determined from the EIS results of G/SiOx/G and NG/SiOx/NG before and after 450 cycles in half-cells.

Electrodes	R_s	R_f	R_{ct}	σ^{-2} [a]
G/SiO_x/G	4.04	-	774.4	2.24×10^{-6}
NG/SiO_x/NG	2.28	-	84.39	8.26×10^{-6}
Cycled G/SiO_x/G	3.14	118.3	517.2	8.89×10^{-7}
Cycled NG/SiO_x/NG	1.56	53.78	197.0	8.73×10^{-5}

[a] Calculated from the Z'-$\omega^{-1/2}$ plots in Figure 5c-d and the diffusion coefficients is proportional to $(\sigma)^{-2}$.

References

1. Hu, J.; Fu, L.; Rajagopalan, R.; Zhang, Q.; Luan, J.; Zhang, H.; Tang, Y.; Peng, Z.; Wang, H. Nitrogen Plasma-Treated Core-Bishell Si@SiO_x@$TiO_{2-\delta}$: Nanoparticles with Significantly Improved Lithium Storage Performance. *ACS Appl. Mater. Interfaces* **2019**, *11*, 27658–27666, doi:10.1021/acsami.9b04415.
2. Mu, G.; Mu, D.; Wu, B.; Ma, C.; Bi, J.; Zhang, L.; Yang, H.; Wu, F. Microsphere-Like SiO_2 /MXene Hybrid Material Enabling High Performance Anode for Lithium Ion Batteries. *Small* **2020**, *16*, 1905430, doi:10.1002/smll.201905430.
3. Majeed, M.K.; Ma, G.; Cao, Y.; Mao, H.; Ma, X.; Ma, W. Metal-Organic Frameworks-Derived Mesoporous Si/SiO_x @NC Nanospheres as a Long-Lifespan Anode Material for Lithium-Ion Batteries. *Chem. Eur. J.* **2019**, *25*, 11991–11997, doi:10.1002/chem.201903043.
4. Fu, R.; Li, Y.; Wu, Y.; Shen, C.; Fan, C.; Liu, Z. Controlling siloxene oxidization to tailor SiO_x anodes for high performance lithium ion batteries. *J. Power Sources* **2019**, *432*, 65–72, doi:10.1016/j.jpowsour.2019.05.071.
5. Yu, Q.; Ge, P.; Liu, Z.; Xu, M.; Yang, W.; Zhou, L.; Zhao, D.; Mai, L. Ultrafine SiO_x/C nanospheres and their pomegranate-like assemblies for high-performance lithium storage. *J. Mater. Chem. A* **2018**, *6*, 14903–14909, doi:10.1039/c8ta03987a.
6. Shi, H.; Zhang, H.; Li, X.; Du, Y.; Hou, G.; Xiang, M.; Lv, P.; Zhu, Q. In situ fabrication of dual coating structured SiO/1D-C/a-C composite as high-performance lithium ion battery anode by fluidized bed chemical vapor deposition. *Carbon* **2020**, *168*, 113–124, doi:10.1016/j.carbon.2020.06.053.
7. Hu, G.; Zhong, K.; Yu, R.; Liu, Z.; Zhang, Y.; Wu, J.; Zhou, L.; Mai, L. Enveloping SiO_x in N-doped carbon for durable lithium storage via an eco-friendly solvent-free approach. *J. Mater. Chem. A* **2020**, *8*, 13285–13291, doi:10.1039/d0ta00540a.
8. Zhang, W.; Weng, Y.; Shen, W.; Lv, R.; Kang, F.; Huang, Z.-H. Scalable synthesis of lotus-seed-pod-like Si/SiO_x@CNF: Applications in freestanding electrode and flexible full lithium-ion batteries. *Carbon* **2020**, *158*, 163–171, doi:10.1016/j.carbon.2019.11.092.
9. Jiang, Y.; Liu, S.; Ding, Y.; Jiang, J.; Li, W.; Huang, S.; Chen, Z.; Zhao, B.; Zhang, J. Modification based on primary particle level to improve the electrochemical performance of SiO -based anode materials. *J. Power Sources* **2020**, *467*, 228301, doi:10.1016/j.jpowsour.2020.228301.
10. Xiao, T.; Zhang, W.; Xu, T.; Wu, J.; Wei, M. Hollow SiO_2 microspheres coated with nitrogen doped carbon layer as an anode for high performance lithium-ion batteries. *Electrochim. Acta* **2019**, *306*, 106–112, doi:10.1016/j.electacta.2019.03.109.
11. Wang, L.; Zhu, X.; Tu, K.; Liu, D.; Tang, H.; Li, J.; Li, X.; Xie, Z.Z.; Qu, D. Synthesis of carbon-SiO_2 hybrid layer @ SiO_2 @ CNT coaxial nanotube and its application in lithium storage. *Electrochim. Acta* **2020**, *354*, 136726, doi:10.1016/j.electacta.2020.136726.
